# Fatigue in Cancer: A Review of Literature

**DOI:** 10.4103/0973-1075.53507

**Published:** 2009

**Authors:** Vijayakumar Narayanan, Cherian Koshy

**Affiliations:** Department of Oncology, St. Gregorios Medical Mission Hospital, Parumala, Pathanamthitta, India; 1 Department of Palliative Care, Regional Cancer Centre, Thiruvanathapuram, Kerala, India

**Keywords:** Chemotherapy, Fatigue, Palliation, Radiotherapy

## Abstract

Fatigue is a common symptom of advanced cancer limiting one's activity and affecting the quality of life. It is a multidimensional symptom complex with subjective and objective components. Hence, its definition and assessment seems arbitrary, incomplete, and elusive. Components of fatigue often merge with other ‘disease states’ as anemia, depression and so on, compounding difficulty to assess it separately. Fatigue has a high prevalence rate, and lasts longer in chronic diseases like cancer. Its association with treatment modalities like chemotherapy, radiotherapy alongside the primary disease process makes it seemingly ubiquitous in many cases. Systemic manifestation of cancer causes excess demand on body resources on cell repair, uncontrolled growth with metabolite accumulation causing fatigue. Co-morbid conditions of organic and psychological nature causes fatigue. There are many assessment tools for fatigue with different uses and objectives, simple and reproducible tools like Brief Fatigue Inventory, Edmonton Symptom assessment scale seem feasible in everyday practice. Management of fatigue is not straightforward and rewarding. Although treatment of cause appears to be an attractive option, it is not possible in all cases. Therapeutic agents targeting cytokine load is in early stages of study and available results are not favorable. Specific measures aimed at pain relief, prevention/treatment of sepsis, management of depression, avoidance of drugs causing fatigue, restoring the metabolic profile are important. Methyl phenidate, megestrol, and modafinil are some drugs with promising effect to treat fatigue, though confirmatory studies are yet to be established. Non-pharmacological methods are also helpful. Forewarning patients on upcoming fatigue, active regular exercise, and stress management are some of them. Fatigue being a multidimensional entity, single mode of therapy is insufficient. Combined modality tailored to individual patient need and understanding may be the right way to battle this ill-understood symptom. This review article examines the etiopathogenesis and management strategies of fatigue in cancer.

## INTRODUCTION

Fatigue is recognized as a common state in palliative care and patients with advanced cancer experience it as the most distressing symptom affecting their quality of life.[1,2,3] Patients characterize fatigue as a feeling of overwhelming exhaustion and lack of energy and enthusiasm. Problems with this symptom is experienced from many months to years following completion of the treatment.[4]

A significant proportion of disease free survivors report disruptive fatigue levels years after their treatment.[5] Fatigue is reported rarely and treatment options or strategies of management are discussed infrequently. Many physicians consider fatigue a ‘standard norm’ of the treatment or disease, on which nothing can be done, and hence to be endured.[6]

Somehow, cancer-related fatigue was downplayed or overlooked for years. Less than 50% of cancer patients discussed the options of treatment of the fatigue symptoms with their oncologist, and only 27% recommended any form of treatment.[7,8] It is poorly understood and very poorly managed. Patient with this long-standing symptom is usually left unsupported and isolated causing a gradual erosion of their already depleted coping repertoire.

## DEFINITIONS

The elusiveness of the condition is evident in its description itself. Fatigue has been presented as a concept, a construct, and as a definition.

A formally accepted definition of fatigue is not established due to complexity of this condition. Most definitions are but useful description or guidelines. National Comprehensive Cancer Network defines cancer-related fatigue as a ‘distressing persistent subjective sense of tiredness or exhaustion related to cancer or cancer treatment that is not proportional to recent activity and interferes with usual functioning.’ (www.nccn.org/physician_gls/PDF/fatigue.pdf, access date 26 April 2009)

The definition according to Oncologic Nursing society is more descriptive. “Fatigue is a feeling of debilitating tiredness or total lack of energy that can last for days, weeks or months; commonly caused by anaemia, fatigue is the side effect of chemotherapy that affects patients the most –more than nausea, pain or depression; symptoms include feeling weak or worn out, having difficulties in climbing stairs, walking short distances and performing simple daily tasks; proper nutrition, light exercise, short naps and medications may help alleviate the fatigue (http://www.cancersymptoms.org/glossary.shtml, access date 26 April 2009).

A concept analysis defines fatigue as a subjective, unpleasant condition which incorporates total body feelings ranging from tiredness to exhaustion creating an unrelenting overall condition that interferes with the individual's ability to function to their normal activity.[9] In contrast to tiredness, subjective fatigue has to be perceived as unusual, abnormal or excessive whole body tiredness, disproportionate to or unrelated to activity or exertion, as suggested by Piper.[10] Fatigue is described as an overwhelming sustained sense of exhaustion and decreased capacity of physical and mental work that is not relieved by rest.[11] International Classification of Diseases (ICD-10)[12] has put forward criteria for identifying cancer related fatigue [Box 1]. This has been set to define the fatigue syndrome in all stages of cancer, from ongoing treatment to advanced stages of the disease as well as to survivorship. The limitation of these criteria is that it cannot be used in defining fatigue in palliative setting, where the occurrence of co-morbid psychological illness is very high.[13,14] The multiplicity of these definitions point to the fact that fatigue is a multidimensional and subjective phenomenon with physical, emotional cognitive, and behavioral dimensions.[9,15]

**Box 1:** Definition of fatigue in the International classification of diseases (ICD -10)[12]

A1 and at least five out of A2–A11 have been present for most days in at least two consecutive weeks in the past month
**A**
A1-Significant fatigue diminished energy or increased need to rest disproportionate to any recent change in activity level
A2-generalized weakness, limb heaviness
A3-diminished concentration or attention
A4-Decreased motivation or interest to engage in usual activities
A5-Insomnia or hypersomnia
A6-Experience of sleep as un-refreshing or non restorative
A7-Perceived need to struggle to overcome inactivity
A8-Marked emotional reactivity (such as sadness, frustration, irritability) to feeling fatigued
A9-Difficulty completing daily tasks attributed to feeling fatigued
A10-Perceived problem with short-term memory
A11-Post exertional malaise lasting several hours
**B**
The symptoms cause clinically significant distress or impairment in social, occupational, or other important areas of functioning
**C**
Evidence from history, physical examination, or laboratory findings that symptoms are a consequence of cancer or cancer treatment
**D**
Symptoms are not primarily a consequence of co-morbid psychiatric disorders such as major depression, somatization disorder, somatoform disorder, or delirium.

The European association of Palliative Care (EAPC) Research steering committee recently put forward a working definition for fatigue as ‘Fatigue is a subjective feeling of tiredness weakness or lack of energy.’[16]

## EPIDEMIOLOGY

The prevalence of fatigue in palliative care setting is in the range of 48-78%.[17] Consistently lower levels of fatigue were observed in healthy controls over cancer patients when standardized instruments were used.[18] In patients who receive chemotherapy, the prevalence is between 75 and 90% and this has a linear increase with progression of treatment. In case of radiation therapy, it is 65% and it does not increase in most cases while on treatment.[19] Long-term survivors of cancer (17-56%) experience fatigue lasting for months after cessation of their treatment with a resultant compromise in the quality of life.[20] Fatigue is commonly associated with other chronic illnesses also. Among elderly people with chronic illnesses, the prevalence is estimated to be in the range of 47-99%.[21] Multiple sclerosis,[22] heart failure,[23] chronic obstructive pulmonary diseases,[24] HIV/AIDS,[25] end stage renal diseases,[26] systemic lupus erythematosus,[27] and rheumatoid arthritis[28] are some of the major chronic illnesses in which fatigue is felt as a troublesome symptom. It should be noted that fatigue is only one dimension of cancer-related asthenia, which is characterized by easy tiring and reduced sustainability of performance, generalized weakness resulting in reduced ability to initiate movement, mental fatigue characterized by poor concentration, impaired memory, and emotional lability.[29]

## CLASSIFICATION AND ETIOPATHOGENEIS

Little is known about the pathophysiology of fatigue in advanced cancer. Patients experience fatigue due to a variety of reasons throughout their cancer journey. The EAPC expert group suggested a differentiation between primary and secondary fatigue. The former is probably related to high cytokine load and the latter is from cancer treatment or concurrent diseases or syndromes.[16] High cytokine content have been found in patients undergoing chemotherapy or radiation treatment. Levels are high in cancer survivors also.[30,31] Cytokine related primary fatigue has central and peripheral components. Changes in the hypothalamo-pituitary-adrenal axis and neuronal system controlling arousal and fatigue constitute the central component, whereas altered muscular metabolism leading to energy imbalance and resultant fatigue is the peripheral component.[32]

It is found that the diurnal rhythm of cortisol secretion is upset in a subtle manner in breast cancer patients. Evening levels of cortisol was found to be comparatively low in these patients than normal population.[33] Hypothalamo-pituitary–adrenal axis was deregulated and this causes changes in circadian rhythm. This is another possible cause for excessive primary fatigue in cancer patients. Cancer, being a systemic disease, puts excessive demands on scarce body resources, for molecular and cellular repair, which is mirrored by an increased level of fatigue.[34] Co-morbid conditions add up to this drain.

Another theory is that the systemic effects of cancer treatment causing accumulation of metabolites as a result of normal tissue damage give rise to fatigue.[35] A wide range of physiological problems contributing to fatigue in advanced cancer is given in Table 1.

**Table 1:** Physiological disorders and associated cause for fatigue in cancer[36]

Physiological disorders	Associated problems
Decreased oxygen carrying capacity	Heart disease, lung cancer, anemia
Metabolic disorders	Liver metastasis
Hypokalemia	Dehydration
Hypophosphatemia	Diarrhea
Hypocalcemia	Renal damage
Hypomagnesemia	Hyperthyroidism, effects of medication
Nutritional disorder	Malnutrition
Anorexia	Mucositis
Cachexia	Gastrointestinal symptoms
Endocrine and hormonal disturbances	Diabetes, low testosterone levels
Disruption of central nervous system functioning	Brain tumors, toxicity from cytotoxic treatment
Immunological disorders	Neutropenia as a result of cancer treatment

Fatigue is closely associated with anemia in cancer patients. Erythropoietin secretion is inhibited because of high levels of cytokines. Decrease in levels of fatigue was observed in patients treated with erythropoietin.[37] However, the association between hemoglobin and fatigue is weak and it is postulated that the impairment in the function of hemoglobin in cancer is the reason for fatigue.[38]

The psychosocial factors associated with fatigue are well documented.[39,40] The conceptualization of fatigue as a consequence of inefficient coping strategies and prolonged stress response is important as it outlines the importance of reinforcement of active or passive coping repertoire in the management of fatigue.[41] The relationship between fatigue and low mood is an established entity.[42] Fatigue is closely related to other symptoms also. It produces a combination of physiological and psychological manifestations and should be considered as a complex multifactorial symptom.

## PREDICTIVE FACTORS

Several factors play in the occurrence of fatigue; however, no specific predictive factors have been identified in the literature. Age is considered a predictive factor though the evidence is conflicting. Younger patients, less than 34 years do better than older patients.[43] Similarly, men over 75 years of age were found to experience 11 times more fatigue than their younger counterparts.[44]

Among women with breast cancer, younger age group was more vulnerable to fatigue, probably due to the aggressiveness of their treatment.[45]

A pre-treatment fatigue level is an important factor. There is evidence that the patients experience fatigue even before their cancer diagnosis.[46]

## ASSESSMENT

Fundamental to the management of fatigue is its comprehensive assessment. Recent years witnessed a quantum leap in fatigue research. Definition and validation of tools pose questions as they are from different patient populations.[47] Assessment tools and definitions of fatigue is crippled with semantic problems as well, for the word *‘fatigue’* is inherent only in French and English languages, and not in other European languages.[16]

Moreover, since fatigue is clearly a multidimensional symptom that is influenced by a number of factors, a wider assessment of other symptoms like pain and psychological morbidity is also necessary while planning an intervention strategy. Several tools are available for clinical assessment of fatigue. In fatigue research, since the aims of study are varied, a detailed and lengthy assessment is mandatory. Fatigue assessment questionnaire,[48] multidimensional fatigue inventory,[49] functional assessment of chronic illness therapy-fatigue (FACIT-F).[50] Piper fatigue scale (revised)[51] are some of the specific instruments for this purpose. Subjective fatigue rating is the most clinically relevant tool. Brief fatigue inventory,[18] Edmonton symptom assessment scale[52,53] are two important tools and are highly valuable for their simplicity and reproducibility. It is imperative to assess the baseline fatigue levels irrespective of whether a simple sliding score of 0-10 or a more detailed tool is used. Consistent evaluation of other symptoms like pain, breathing difficulty, depression, and a detailed analysis of parameters also should be part of fatigue assessment. Any assessment of fatigue is dependent on the subjective evaluation of the symptom by the patient. Since palliative care focuses on the subjective condition of a patient, the subjective assessment should be the indicator for treatment. Clinical help is sought only if self-assessment is not feasible in situations like severe cognitive impairment. Self-assessment is not feasible with children as well. No assessment tools have been developed so far for children. Hence, the recommendation is that the assessment of children should be made by the parents or by staff using behavioral observations such as changes in sleep–wake time, frequent dozing, and continuous lack of interest or concentration difficulties.[16]

## MANAGEMENT STRATEGIES

Cancer related fatigue is one of the poorly attended symptoms. Inadequate skills and lack of awareness on the symptoms among the physicians can be suggested as reason, apart from under reporting.[16]

### Treatment of primary fatigue

Pharmacological agent s that are targeting excessive cytokines have been an area of interest and a variety of such agents like Thalidomide, Pentoxyfyllin, and Rolipram are currently under investigation. However, early reports are not favorable.[54,55] Treatment of the underlying cause, if any, is the mainstay in the management of the symptom.

The opioid dose should be individually tailored and dose modifications should be made for an optimum pain reduction. WHO analgesic ladder is the guideline.[56] Specific measures for the management of anemia in elderly include replenition of iron, folate and/or vitamin B12. If the cause cannot be identified or if the patient refuses work up, symptomatic management can be carried out. Red blood transfusion is the single most effective and cheap solution. Erythropoietin improves anemia in elderly in conditions like chronic renal diseases.[57] The patient may have poor marrow reserve if a substantial portion of bone marrow is affected. Correction of anemia in advanced cancer patients at terminal stages has limited impact on the intensity of fatigue.[58]

Metabolic disorders should be corrected. Electrolyte, hormonal imbalances are to be managed and endocrine problems should be addressed. Adequate hydration and treatment of sepsis is to be attempted. Nutritional supplementation may be tried. Megestrol acetate in the dose range of 160-480 mg per day was shown to improve appetite in many trials.[59,60] But its role in alleviating fatigue is not proven.[60,61] Treatment of depression with antidepressants may alleviate fatigue in cancer patients.[62] Selective serotonin reuptake inhibitors (SSRIs) and atypical antidepressants are preferable over tricyclics, as they cause less side effects.[63] Any medications that contribute to fatigue should be discontinued. Box 2 shows the list of main causes of primary fatigue and their management.

**Box 2:** Main causes of primary fatigue and their management

Anaemia	Erythropoietin/transfusion
Infection	Antibiotics
Fever	Antipyretics
Pain	Analgesics
Dehydration	Hydration
Electrolyte imbalance	Biphosphonates/substitution
Cachexia	Nutrition/anabolic agents
Hypothyroidism hypogonadism	Hormone substitution
Depression	Antidepressant medications
Sleep disturbance	Sleep hygiene/ sedative/co-medication/reduce/rotate drugs

### Treatment of secondary fatigue

#### Pharmacological

Methyl phenidate is an amphetamine derivative acting on synaptic monoamine receptors and facilitates the release of catecholamine release. The onset of action is fast and it has a half life of ~2 hrs. It is metabolized in liver and excreted through kidneys. It is particularly useful in depression in palliative care settings.[64,65] Its value in opioid sedation is also well established.[66,67] The usual prescribed dosage is 5-10 mg orally in morning which can be titrated up to 40-60 mg per day with dose limiting side effects like loss of appetite, slurring of speech, nervousness, and cardiac symptoms. Role of methyl phenidate in relieving cancer related fatigue is still under exploration as the available results are not favorable.[68]

Megestrol acetate, in the dose range of 480-800 mg has been FDA approved for cachexia related to HIV/AIDS and cancer related-fatigue. The drug improves appetite, increases activity, and contributes to overall well being in advanced cancer patients,[59] though the precise mechanism is not known. Hypertension, sweating, hot flashes, weight gain, mood swings, and GIT upset are the common side effects.

The centrally acting acetyl cholinesterase inhibitor drug Donepezil was found to be effective in opioid induced sedation.[69] However, the protracted half life of 70 hours makes its use problematic in palliative care setting.

Corticosteroids such as methyl prednisolone or dexamethasone are recommended for relieving fatigue in short periods.[70,71] Infection, mood swings, insomnia, myalgia, and elevation of blood glucose are the side effects.

Modafinil, a GABA inhibitor has been shown to be effective in relieving fatigue in many chronic conditions like HIV/AIDS, multiple sclerosis, Parkinson's disease.[72–74] Its use in cancer related-fatigue is under study. The recommended dose is 200-400 mg per day. The adverse effects are GIT upset, headache, dizziness, and rhinitis. Cardiac dysaarrythmia is a serious adverse effect, but very rare.

#### Non-pharmacological

Forewarning patients about the symptoms of fatigue and providing information on strategies to alleviate it can provide some relief and reduces the anxiety of unexpected symptoms. If the patients are asked to discuss about their fatigue, that in turn helps them to make the symptom more tangible and may reduce the uncertainty of its occurrence and associated distress.[75] Exercise has proven to be effective in alleviating fatigue symptoms. In a randomized study, self-paced incremental 20-30 minutes walk four to five times a week resulted in better physical functioning, lower symptom intensity, and lower fatigue levels than those in the control group.[76] Fatigued patients mostly tend to rest, nap or sleep[77] which lead to more night time waking up and increased fatigue levels. These are passive strategies, and fail to decrease cancer-related fatigue.[78] Energy conservation is in fact a de-conditioning that creates a vicious cycle of fatigue and further de-conditioning, which later leads to disability.[79]

Significant improvement in affective state and alleviation of pain was observed in patients who underwent a group exercise therapy twice a week, each session lasting for 50 minutes, for six weeks.[80]

Stress management techniques help to reduce the anxiety associated with fatigue symptoms. Difficulty in thinking and concentration was found to be improved if the patient focuses on the recreational activities for 20-30 minutes three times a week.[81] Brief psycho-educational group intervention focusing on active coping strategies and physical activity was found to be beneficial to cancer survivors after breast cancer treatments.[82] Individualized counseling has a major role in reducing distress and fatigue.[83]

## CONCLUSION

The complexity of fatigue warrants a transdisciplinary approach for an effective management. It includes pharmacological interventions, psycho education, individual exercises, couns eling, and information. Treatment interventions have to be individualized and each patient should have his or her own plans to combat fatigue. This needs sustained effort. Prompt symptom assessment and early intervention is the key to a successful management plan. Fatigue experienced by a patient at the close of life is a normal phenomenon, and a vigorous attempt for reversal in the final hours may not be appropriate as it may cause a re-entry into the world of suffering. Hence, a judicious approach is most necessary in dealing with cancer-related fatigue.

## References

[1] National comprehensive cancer net work, American Cancer Society (2008) Cancer related fatigue and anemia, treatment guidelines for patients, Version I.

[2] Ahlberg K, Ekman T, Gaston-Johansson F, Mock V (2003). Assessment and management of cancer-related fatigue in adults. Lancet.

[3] Portoney RK, Itri LM (1994). Cancer related fatigue: Guidelines for evaluation and management. Oncologist.

[4] Wagner LI, Cella D (2004). Fatigue and cancer: Causes, prevalence and treatment approaches. Br J Cancer.

[5] Morrow GR, Andrews PL, Hickok JT, Roscoe JA, Matteson S (2002). Fatigue associated with cancer and its treatment. Support Care Cancer.

[6] Radbruch L, Strasser F, Elsner F, Gonçalves JF, Løge J, Kaasa S (2008). Fatigue in palliative care patients. Palliat Med.

[7] Vogelzang NJ, Breitbart W, Cella D, Curt GA, Groopman JE, Horning SJ (1997). Patient, caregiver and oncologist perceptions of cancer -related fatigue: Results of a tri-part assessment survey. Semin Haematol.

[8] Stone P, Richardson A, Ream E, Smith AG, Kerr DJ, Kearney N (2000). Cancer related fatigue: Inevitable, unimportant and untreatable? Results of a multi-centre patient survey. Ann Oncol.

[9] Reem E, Richardson A (1996). Fatigue: A concept analysis. Int J Nurs Stud.

[10] Piper B, Carrieri V, Lindsey A, West C (1993). Pathophysiological phenomena in Nursing: Human responses to illness.

[11] Carpentio LJ, Carpentio LJ (1995). Fatigue. Nursing diagnosis: Applications to clinical practice.

[12] Cella D, Davis K, Breibart W, Curt G (2001). Cancer-related fatigue: Prevalence of proposed diagnostic criteria in a United States sample of cancer survivors. J Clin Oncol.

[13] Murphy H, Alexander S, Stone P (2006). Investigation of diagnostic criteria for cancer-related fatigue syndrome in patients with advanced cancer: A feasibility study. Palliat Med.

[14] Fernandez R, Stone P, Andrews P, Morgan R, Sharma S (2006). Comparison between fatigue, sleep disturbance and circadian rhythm in cancer inpatients and healthy volunteers: evaluation of diagnostic criteria for cancer related fatigue. J Pain Symptom Manage.

[15] Smets EM, Garssen B, Bonke B, de Haes JC (1995). The multidimensional fatigue Inventory: Psychometric qualities of an instrument to assess fatigue. J Psychsom Res.

[16] Radbruch L, Strasser F, Elsner F, Gonçalves JF, Løge J, Kaasa S (2008). Fatigue in palliative care patients: An EAPC approach. Palliat Med.

[17] Smets EM, Garssen B, Schuster-Uittenhoeve AL, de Haes JC (1993). Fatigue in cancer patients. Br J Cancer.

[18] Mendoza TR, Wang XS, Cleeland CS, Morrissey M, Johnson BA, Wendt JK (1999). The rapid assessment of fatigue severity in cancer patients. Cancer.

[19] Stone P (2002). The measurement, causes and effective management of cancer-related fatigue. Int J Palliat Nurs.

[20] Lawrence DP, Kupelnick B, Miller K, Devine D, Lau J (2004). Evidence report on the occurrence, assessment and treatment of fatigue in cancer patients. J Natl Cancer Inst Monogr.

[21] Stone P, Richards M, A'Hern R, Hardy J (2000). A study to investigate the prevalence, severity and correlates of fatigue among patients with cancer in comparison with a control group of volunteers without cancer. Ann Oncol.

[22] Krupp LB (2003). Fatigue in multiple sclerosis. CNS Drugs.

[23] Goodlin SJ (2005). Palliative care for end stage heart failure. Curr Heart Fail Rep.

[24] Elkington H, White P, Addington-Hall J, Higgs R, Edmonds P (2005). The health care needs of chronic obstructive pulmonary disease patients in the last year of life. Palliat Med.

[25] Breitbart W, McDonald MV, Rosenfeld B, Monkmann ND, Passik S (1998). Fatigue in ambulatory AIDS patients. J Pain Symptom Manage.

[26] Cardenas DD, Kutner NG (1982). The problem of fatigue in dialysis patients. Nephron.

[27] Wysenbeek AJ, Leibovici L, Weinberger A, Guedj D (1993). Fatigue in systemic lupus erythematosus. Br J Rheumatol.

[28] Wolfe F, Hawley DJ, Wilson K (1996). The prevalence and meaning of fatigue in rheumatic disease. J Rheumatol.

[29] Woof R, Faull C (1998). Asthenia cachexia and anorexia. Handbook of Palliative care.

[30] Meyers CA, Albitar M, Estey E (2005). Cognitive impairment, fatigue and cytokine levels in patients with acute myelogenous leukaemia or myelodysplastic syndrome. Cancer.

[31] Greenberg DB, Gray JL, Mannix CM, Eisenthal S, Carey M (1993). Treatment- related fatigue and serum Interleukin-I levels in patients during external beam irradiation for prostate cancer. J Pain Symptom Manage.

[32] Gulstein HB (2001). The biologic basis of fatigue. Cancer.

[33] Bower JE, Ganz PA, Dickerson SS, Peterson L, Aziz N, Fahey JL (2005). Diurnal cortisol rhythm and fatigue in breast cancer survivors. Psychoneuroendocrinology.

[34] Beach P, Siebeneck B, Buderer NF, Ferner T (2001). Relationship between fatigue and nutritional status in patients receiving radiation therapy to treat lung cancer. Oncol Nurs Forum.

[35] Glause A (1998). Fatigue in patients with cancer: Analysis and assessment, vol.145. Recent results in cancer research.

[36] Moore G, Hayes C, Watkins- Bruner D, Moore-Higgs G, Haas M (2001). Maintenance of comfort (fatigue and pain). Outcomes in radiation therapy: multidisciplinary management.

[37] Cella D, Dobrez D, Glaspy J (2003). Control of cancer-related anemia with erythropoetic agents: A review of evidence for improved quality of life and clinical outcomes. Ann Oncol.

[38] Stone PC, Abdul Wahab A, Gibson JS, Wright RJ, Andrews PL (2005). Fatigue in cancer patients is not related to changes in oxyhemoglobin dissociation. Support Care Cancer.

[39] Curran SL, Beacham AO, Andrykowsky MA (2004). Ecological momentary assessment of fatigue following breast cancer treatment. J Behav Med.

[40] Servaes P, Verhagen C, Bleigenberg G (2002). Fatigue in cancer patients during and after treatment: prevalence, correlates and interventions. Eur J Cancer.

[41] Gelinas C, Fillion L (2004). Factors related to persistent fatigue following completion of breast cancer treatment. Oncol Nurs Forum.

[42] Blesch KS, Paice JA, Wickham R, Harte N, Schnoor DK, Purl S (1991). Correlates of fatigue in people with lung and breast cancer. Oncol Nurs Forum.

[43] Fobair P, Hoppe RT, Bloom J, Cox R, Varghese A, Spiegel D (1986). Psychosocial problems among survivors of Hodgkin's disease. J Clin Oncol.

[44] Faithful S (2000). Supportive care in radiotherapy: Evaluating the potential contribution of nursing: Institute of Cancer Research.

[45] Woo B, Dibble SL, Piper BF, Keating SB, Weiss MC (1998). Differences in fatigue by treatment methods in women with breast cancer. Oncol Nurs Forum.

[46] Piper B, Dodd M (1991). Self initiated fatigue interventions and their perceived effectiveness. Oncol Nurs Forum.

[47] Meek PM, Nail LM, Barsevick A, Schwartz AL, Stephen S, Whitmer K (2000). Psychometric testing of fatigue instruments for use with cancer patients. Nursing Res.

[48] Glaus A (1998). Fatigue in patients with cancer: Analysis and assessment. Cancer Res.

[49] Smets EM, Garssen B, Bonke B, De Haes JC (1995). The multi-dimensional fatigue inventory (MFI) psychometric qualities of an instrument to assess fatigue. J Psychosom Res.

[50] Cella D (1997). Manual of the functional assessment of chronic illness therapy (FACIT). Measurement system. Evanston, Ill. Center on Outcomes, Research and Education (CORE).

[51] Piper BF, Dibble SL, Dodd MJ, Weiss MC, Slaughter RE, Paul SM (1998). The revised Piper fatigue scale: Psychometric evaluation in women with breast cancer. Oncol Nurs Forum.

[52] Bruera K, Kuehn N, Miller MJ, Selmser P, Macmillan K (1991). () The Edmonton symptom assessment system (ESAS). J Palliat Care.

[53] Chang VT, Hwang SS, Feuerman M (2000). Validation of the Edmonton symptom assessment scale. Cancer.

[54] Gordon JN, Trebble TM, Ellis RD, Duncan HD, Johns T, Goggin PM (2005). Thalidomide in the treatment of cancer cahexia: A randomised placebo controlled trial. Gut.

[55] Goldberg RM, Loprinzi CL, Mailliard JA, O'Fallon JR, Krook JE, Ghosh C (1995). Pentoxyfyllin for treatment of cancer anorexia and cachexia? A randomised, double blind, placebo-controlled trial. J Clin Oncol.

[56] World Health Organisation (1996). Cancer Pain relief.

[57] Drueke TB, Locatelli F, Clyne N (2006). Normalisation of haemoglobin level in patient with chronic kidney disease and anaemia. N Engl J Med.

[58] Conill C, Verger E, Henriquez I, Saiz N, Espier M, Lugo F (1997). Symptom prevalence in the last week of life. J Pain Symptom Manage.

[59] Bruera E, Ernst S, Hagen N, Spachynski K, Belzile M, Hanson J (1998). Effectiveness of megestrol acetate in patients with advanced cancer. Cancer Prev Control.

[60] De Conno F, Martini C, Zecca E, Balzarini A, Venturino P, Groff L (1998). Megestrol acetate for anorexia in patients with far advanced cancer: A double blind controlled clinical trial. Eur J Cancer.

[61] Westman G, Bergman B, Albertsson M, Kadar L, Gustavsson G, Thaning L (1999). Megestrol acetate in advanced, progressive, hormone insensitive cancer: Effects on the quality of life: A placebo controlled, randomised, multicentric trial. Eur J Cancer.

[62] Lander M, Wilson K, Chochinov HM (2000). Depression and the dying older patient. Clin Geriatr Med.

[63] Kasper S, de Swart H, Friis Anderson H (2005). Escitalopram in the treatment of depressed elderly patients. Am J Geriatr Psychiatry.

[64] Sarhill N, Walsh D, Nelson KA, Homsi J, Le Grand S, Davis MP (2001). Methyl phenidate for fatigue in advanced cancer: A prospective open-label private study. Am J Palliat Care.

[65] Homsi J, Walsh D, Nelson KA, Legrand S, Davis M (2000). Methyl phenidate for depression in hospice practice: A case series. Am J Palliat Care.

[66] Wilwerding MB, Loprinzi CL, Mailliard JA, O'Fallon JR, Miser AW, van Haelst C (1995). A randomised, cross over evaluation of methyl phenidate in cancer patients receiving strong narcotics. Support Care Cancer.

[67] Bruera E, Chadwick S, Brenneis C, Hanson J, Mac Donald RN (1987). Methyl phenidate associated with narcotics for the treatment of cancer pain. Cancer Treat Rep.

[68] Bruera E, Valero V, Driver L, Barnes EA, Willey J, Shen L, Palmer JL (2003). Patient controlled methyl phenidate for the management of fatigue in patients with advanced cancer: A double blind randomised placebo -controlled trial. J Clin Oncol.

[69] Slatkin NE, Rhiner M (2003). Treatment of opiate-related sedation: Utility of the cholinesterase inhibitors. J Support Oncol.

[70] Bruera E, Roca E, Cedaro L, Carraro S, Chakon R (1985). Action of oral methyl prednisolone in terminal cancer patients. Cancer Treat Rep.

[71] Hardy JR, Rees E, Ling J, Burman R, Feuer D, Broadley K (2001). A prospective survey for the use of dexamathasone on a palliative care unit. Palliat Med.

[72] Rabkin JG, Mc Elhiney MC, Rabkin R, Ferrando SJ (2004). Modafinil treatment for fatigue in HIV + patients: a pilot study. J Clin Psychiatry.

[73] Stankoff B, Waubant E, Confavreux C, Edan G, Debouverie M, Rumbach L (2005). Modafinil for fatigue in Multiple Sclerosis: a randomised placebo-controlled double blind study. Neurology.

[74] Ondo WG, Fayle R, Atassi F, Jankovic J (2005). Modafinil for day time somnolence in Parkinson's disease: double blind placebo-controlled parallel trial. J Neurol Neurosurg Psychiatry.

[75] Krishnasamy M (1997). Exploring the nature and impact of fatigue in advanced cancer. Int J Palliat Nurs.

[76] Mock V, Hassey-Dow K, Meares C, Grimm PM, Dienemann JA, Haisfield-Wolfe ME (1997). Effects of exercise on fatigue, physical functioning and emotional distress during radiation therapy for breast cancer. Oncol Nurs Forum.

[77] Pearce S, Richardson A (1994). Fatigue and cancer: a phenomenological study. J Cancer Nurs.

[78] Faithfull S, Faithfull S, Weils M (2003). Fatigue and radiotherapy. Supportive care in Radiotherapy.

[79] Winningham ML (2001). Strategies for managing cancer-related fatigue syndrome. Cancer.

[80] Oldervol LM, Loge JH, Paltiel H, Asp MB, Vidvei U, Wiken AN (2006). The effect of a physical exercise programme in palliative care. J Pain Symptom Manage.

[81] Cimprich B (1993). Development of an intervention to restore attention in cancer patients. Cancer Nursing.

[82] Fillion L, Gagnon P, Leblond F, Gélinas C, Savard J, Dupuis R (2008). A brief intervention for fatigue management in breast cancer survivors. Cancer Nursing.

[83] Trijsberg R, van Knippenberg F, Rijpma S (1992). Effects of psychological treatment on cancer patients: a critical review. Psychosom Med.

